# Research transparency promotion by surgical journals publishing randomised controlled trials: a survey

**DOI:** 10.1186/s13063-020-04756-7

**Published:** 2020-10-01

**Authors:** N. Lombard, A. Gasmi, L. Sulpice, K. Boudjema, F. Naudet, D. Bergeat

**Affiliations:** 1 Department of HPB and Digestive Surgery, CHU Rennes, Pontchaillou Hospital, Rennes, France; 2 Rennes 1 University, Rennes, France; 3 Institut NuMeCan (Nutrition Metabolism and Cancer), INSERM 1241, F-35000 Rennes, France; 4 Univ Rennes, CHU Rennes, Inserm, CIC 1414 [(Centre d’Investigation Clinique de Rennes)], F-35000 Rennes, France

**Keywords:** Data sharing, Conflict of interest, CONSORT statement, Randomised controlled trial

## Abstract

**Objective:**

To describe surgical journals’ position statements on data-sharing policies (primary objective) and to describe key features of their research transparency promotion.

**Methods:**

Only “SURGICAL” journals with an impact factor higher than 2 (Web of Science) were eligible for the study. They were included, if there were explicit instructions for clinical trial publication in the official instructions for authors (OIA) or if they had published randomised controlled trial (RCT) between 1 January 2016 and 31 December 2018. The primary outcome was the existence of a data-sharing policy included in the instructions for authors. Data-sharing policies were grouped into 3 categories, inclusion of data-sharing policy mandatory, optional, or not available. Details on research transparency promotion were also collected, namely the existence of a “prospective registration of clinical trials requirement policy”, a conflict of interests (COIs) disclosure requirement, and a specific reference to reporting guidelines, such as CONSORT for RCT.

**Results:**

Among the 87 surgical journals identified, 82 were included in the study: 67 (82%) had explicit instructions for RCT and the remaining 15 (18%) had published at least one RCT. The median impact factor was 2.98 [IQR = 2.48–3.77], and in 2016 and 2017, the journals published a median of 11.5 RCT [IQR = 5–20.75].

The OIA of four journals (5%) stated that the inclusion of a data-sharing statement was mandatory, optional in 45% (*n* = 37), and not included in 50% (*n* = 41).

No association was found between journal characteristics and the existence of data-sharing policies (mandatory or optional). A “prospective registration of clinical trials requirement” was associated with International Committee of Medical Journal Editors (ICMJE) allusion or affiliation and higher impact factors. Journals with specific RCT instructions in their OIA and journals referenced on the ICMJE website more frequently mandated the use of CONSORT guidelines.

**Conclusion:**

Research transparency promotion is still limited in surgical journals. Standardisation of journal requirements according to ICMJE guidelines could be a first step forward for research transparency promotion in surgery.

## Background

Surgical journals have a key role in ensuring transparency, openness, and reproducibility [1] to increase value and reduce waste in the research they publish [2]. Editorial standards promoting transparency are expected when it comes to randomised controlled trials (RCT) because their importance is paramount in drafting guidelines that can impact medical practice worldwide. Surgical interventions are invasive and in some surgical trials, participants may expose themselves to heightened risk with uncertain benefits. This results in an implicit social contract imposing an ethical obligation whereupon the results lead to the greatest possible benefit to society [3].

The latest breakthrough was the adoption of a policy that encourages RCT data sharing and requires a data-sharing statement to be included in the reports of published clinical trials by the International Committee of Medical Journal Editors (ICMJE) [4]. According to the ICJME recommendations, the data-sharing statement must indicate if individual anonymised data will be shared. The statement must clearly specify the start and end date that the data will be available, with whom the data may be shared, the modality of access, and other documents that would be available.

Other aspects of research transparency promotion have been previously promoted, such as registration of the trial [5], adoption of the CONSORT statement [6], and declaration of conflict of interest (COI) [7]. However, transparent practices in the surgical community could be suboptimal, as suggested by the underreporting of COI [8].

If we hypothesise that editors can be the first motivators for research transparency promotion, reviewing current editors’ practices and policies in relation to the transparency of the research is pertinent, before evaluating the evolution of those practices in papers published in surgical journals.

The aim of this study is to describe the surgical journal position statement on data-sharing policies (primary objective) and to describe the key features of their research transparency promotion.

## Methods

The protocol of the survey of surgical journals was registered in the Open Science Framework (OSF) on February 25, 2019 [9].

### Eligibility criteria and journal selection

Two reviewers (NL and AG) used Web of Science to select journals classified in the “SURGICAL” category with a 2017 impact factor higher than 2. Surgical journals were included if there were explicit instructions for clinical trial publication in the instructions for authors, or if they published at least one RCT between 1 January 2016 and 31 December 2018. Two authors (NL and AG) independently extracted the data. Disagreements were resolved by consensus or in consultation with a third reviewer (DB). The list of journals was extracted in December 2018, and the official instructions for authors (OIA) were downloaded on 13–14 January 2019.

Our primary outcome was the existence of a data-sharing policy in the instructions for authors. The following classification was used to describe the types of policies for data sharing: “Inclusion of data-sharing policy mandatory”, “inclusion of data-sharing policy optional” and “no data-sharing policy available”.

Additional details on research transparency promotion were also collected, namely, the existence of a “prospective registration of clinical trials requirement” policy, a “COIs” disclosure requirement, and a specific reference to reporting guidelines such as CONSORT for RCT.

Other variables were also extracted, in order to detail journals features as well as potential predictors for journals’ adherence to research transparency rules. The 2017 journal impact factors were extracted from the Web of Science data base. The number of RCT published in 2016 and 2017 (1/1/16 to 12/31/17) was extracted from PubMed. As described in the protocol, it was originally planned to extract the number of RCT published in 2016, 2017, and 2018, but this was not possible because at the time of data extraction, not all RCT published in 2018 were fully indexed in PubMed. ICMJE “affiliation” was defined as journals referenced at time of data extraction as “Journals stating that they follow ICMJE Recommendations” at: http://www.icmje.org/journals-following-the-icmje-recommendations. Publishing model was classified according to the details described in the instructions to authors referring to the method used to fund publications: either by the authors (open access), reader (subscription to the journal or pay per view), and optional (can be the author, equivalent to open access or the reader).

### Statistical analyses

Analyses of all included journals were performed using R statistical software (http://www.r-project.org/). Continuous data were presented using median and interquartile range (IQR) and compared with the Mann-Whitney *U* test. Categorical data were presented as a percentage and compared with a chi-squared test or a two-sided Fisher’s exact test when Chi-squared test application conditions were not met. Univariate exploratory analyses were performed to explore the associations between journal features and the various transparency policies. For exploratory analyses, publishers were grouped into 3 classes according to the number of journal titles included in this study: “high” with over 20 titles, “middle” with 10 to 20 titles, and “low” with less than 10 titles. The number of RCTs published and the 2017 journal impact factor were separated into quartiles using the quant.cut () function, with following parameters, including the lowest variable and excluded the right. For exploratory analyses, the threshold for statistical significance was set at *P* < 0.05/36 (*P* = 0.0014) with a Bonferroni correction due to multiple comparisons (*n* = 36). Multivariable analysis was not performed due to sparse data (i.e. too small sample size in some groups).

## Results

**Fig. 1:**
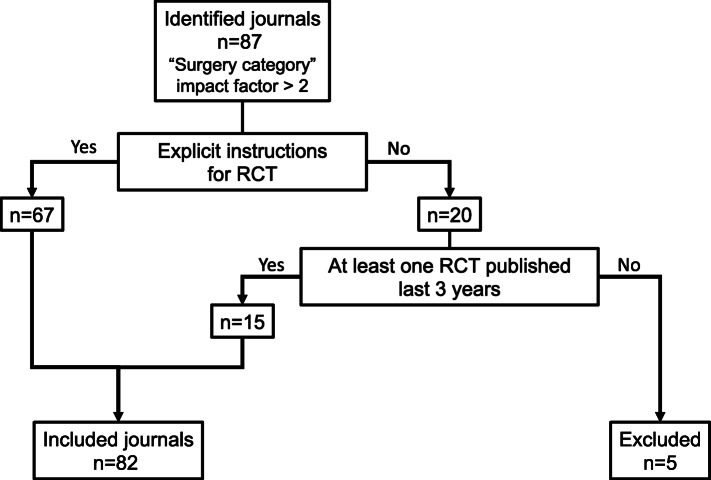
Journal selection process. RCT, randomised controlled trial

Of the 87 surgical journals identified, 82 were included in the analysis: 67 (82%) had explicit instructions for RCT and the remaining 15 (18%) had published at least one RCT between 2016 and 2018 (Fig. 1). The characteristics of these journals are detailed in Table 1. The median impact factor was 2.98 [IQR = 2.48–3.77]. In 2016 and 2017, the journals published a median of 12 RCT [IQR = 5–21]. The publishing model was “optional” in most cases (89%), and America was the principal geographical area of journal editorial committees (56%).

**Table 1:** Journal characteristics and policies

Characteristics of included journals	***n*** = 82
**Explicit instruction(s) for RCT in the OIA**	67 (82%)
**Allude to ICMJE guidelines in the OIA**	63 (77%)
ICMJE affiliation*	35 (43%)
**Publisher**	
Elsevier	25 (30%)
Other	21 (26%)
Springer Nature	13 (16%)
Wiley Online Library	12 (15%)
Wolters Kluwer	11 (13%)
**Geographical area of major editorial committee**	
America	46 (56%)
Europe	26 (32%)
Asia	6 (7%)
Multinational	4 (5%)
**Journal topics**	
Surgery in specialised topics	62 (76%)
Surgery in general topics	20 (24%)
**Publishing model**	
Optional	73 (89%)
Reader	8 (10%)
Authors	1 (1%)
**Number of RCT during the study period**	12 [5–21]
**2017 Journal impact factor**	2.98 [2.48–3.77]

Categorical data are reported with number and (%); continuous data are reported with median and [interval interquartile range (IQR )]

*OIA* official instructions for authors, *ICMJE* International Committee of Medical Journal Editors

*Journals referenced as “Journals stating that they follow the ICMJE Recommendations” on http://www.icmje.org/journals-following-the-icmje-recommendations/

**Fig. 2:**
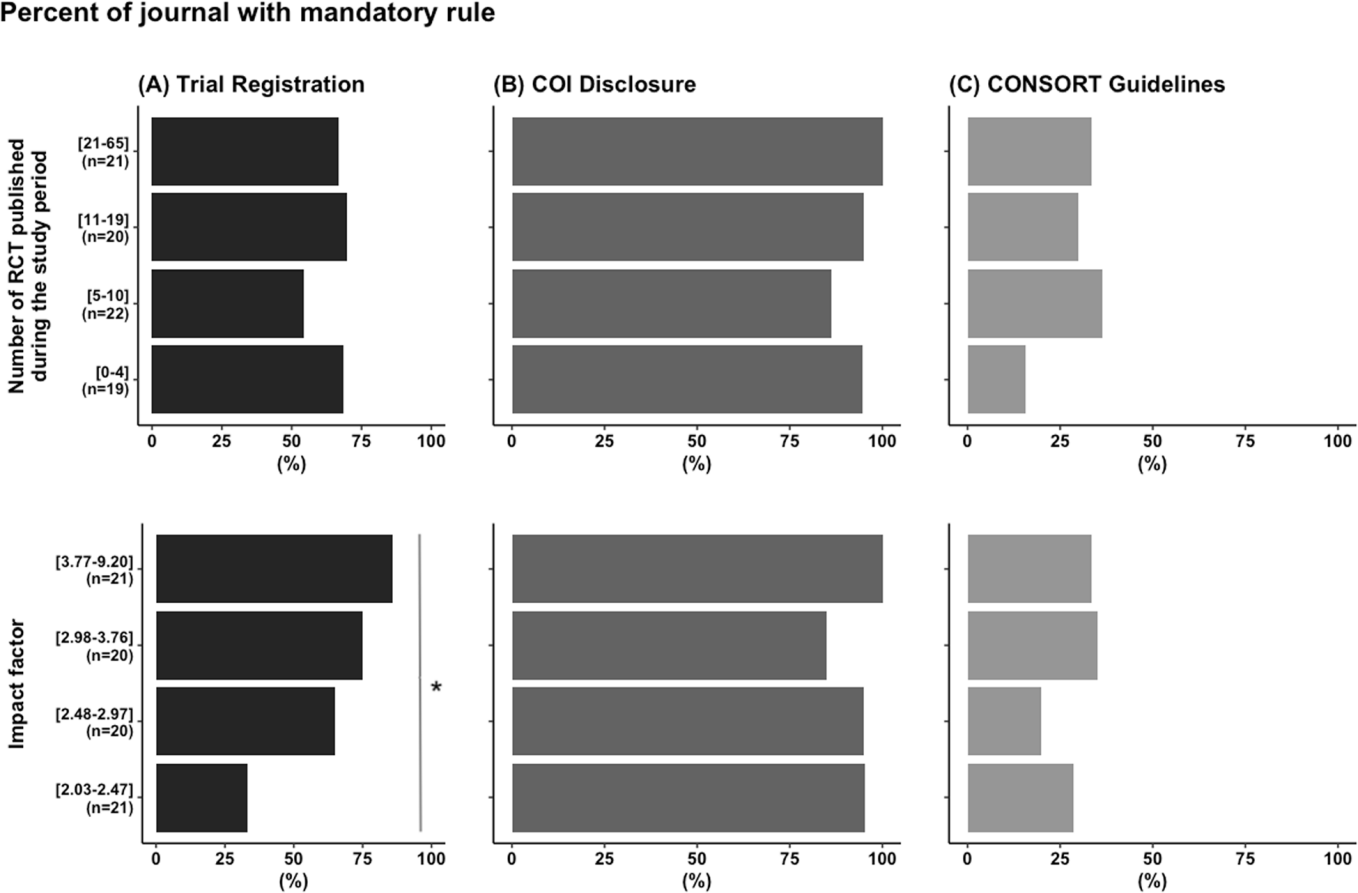
Percent of journal with mandatory rules concerning **a** trial registration, **b** COI disclosure, and **c** CONSORT guidelines according to the number of RCT quartiles published during the study period and to the impact factor quartiles. COI, conflict of interest; RCT, randomised controlled trial. Significant *P* value (*P* = 0.03) is highlight with “*”

Adherence to research transparency rules are detailed in Table 2. Data-sharing statement instructions were present in the OIAs in 41 journals (50%). The OIA for four journals (5%) stated that the inclusion of a data-sharing statement was mandatory, optional in 45% (*n* = 37), and not included in 50% (*n* = 41). COI disclosure was mandatory in 77 journals (94%). A reference to CONSORT guidelines was made in 24 journals (29%). Prospective registration of clinical trials was mandatory in 53 cases (65%). Table 3 details the relationship between data-sharing policies and journal impact factors and the number of RCT published in 2016 and 2017 grouped by quartiles. The association of other research transparency promotion item relationships with journal impact factor and the number of RCT published are illustrated in the Fig. 2. Only the association between journal impact factor and trial registration mandatory rule was statistically significant (*P* = 0.003)**.** Other exploratory analysis of journal features and the different transparency policies are presented in Table 4. As for data-sharing statements, no association was found between journal characteristics and the existence of data-sharing policies (mandatory or optional). A “prospective registration of clinical trials requirement” was associated with ICMJE allusion (*P* < 0.001), ICMJE affiliation (*P* < 0.001), and higher impact factors (*P* < 0.001). Journals with specific RCT instructions in their OIA (*P* = 0.04) and journals referenced on the ICMJE website (*P* = 0.03) more frequently mandated the use of CONSORT guidelines, but those results were not significant using our *P* value threshold (*P* = 0.0014). No other pertinent association was found.

**Table 2:** Adherence to research transparency rules

Adherence to research transparency rules	***n*** = 82
**Data-sharing statement** (primary outcome)	
Yes	41 (50%)
No	41 (50%)
**Inclusion of data-sharing policy**	
Mandatory	4 (5%)
Optional	37 (45%)
Not available	41 (50%)
**Prospective registration of clinical study policies**	
Mandatory	53 (65%)
Optional	29 (35%)
**Consort guideline policies**	
Mandatory	24 (29%)
Optional	58 (71%)
**COI disclosure policies**	
Mandatory	77 (94%)
Optional	5 (6%)

*COI* conflict of interests, *ICMJE* International Committee of Medical Journal Editors

**Table 3:** Relationship between data-sharing policies and journal impact factors and the number of RCT during the study period grouped by quartiles

	Inclusion of data-sharing statement policy				
	Total	Mandatory	Optional	Not available	***P*** value
	*n* = 82	*n* = 4	*n* = 37 (%)	*n* = 41	
**Number of RCT published during the study period**					0.47
[0–4]	19 (23%)	0 (0%)	8 (42%)	11 (58%)	
[5–10]	22 (27%)	1 (5%)	9 (41%)	12 (55%)	
[11–19]	20 (24%)	3 (15%)	9 (45%)	8 (40%)	
[21–65]	21 (26%)	0 (0%)	11 (52%)	10 (48%)	
**2017 journal impact factor**					0.20
[2.03–2.47]	21 (26%)	0 (0%)	13 (62%)	8 (38%)	
[2.48–2.97]	20 (24%)	0 (0%)	9 (45%)	11 (55%)	
[2.98–3.76]	20 (24%)	1 (5%)	9 (45%)	10 (50%)	
[3.77–9.20]	21 (26%)	3 (14%)	6 (29%)	12 (57%)

**Table 4:** Exploratory analysis, association between journal features, and transparency policies with univariate analysis

	Research transparency promotion by surgical journals											
	Data-sharing support			Mandatory prospective registration of clinical study			Mandatory follow-up of CONSORT guidelines			Mandatory conflict of interest disclosure		
	Yes	No	***P*** value	Yes	No	***P*** value	Yes	No	***P*** value	Yes	No	***P*** value
	***n*** = 41	***n*** = 41		***n*** = 53	***n*** = 29		***n*** = 24	***n*** = 58		***n*** = 77	***n*** = 5	
**Explicit instruction (s) for RCT in the OIA**	32 (78)	35 (85)	0.56	53 (100)	14 (48)	**< 0.001**	24 (100)	43 (74)	0.004	63 (82)	4 (80)	1.00
**Allude to ICMJE guidelines in the OIA**	29 (71)	34 (83)	0.29	49 (92)	14 (48)	**< 0.001**	19 (79)	44 (76)	0.97	60 (78)	3 (60)	0.33
ICMJE affiliation*	17 (41)	18 (44)	1	27 (51)	8 (28)	0.07	15 (63)	20 (34)	0.03	34 (44)	1 (20)	0.38
**Journal impact factor**	2.77 [2.38–3.69]	3.12 [2.64–3.86]	0.28	3.29 [2.69–4.09]	2.53 [2.29–3.00]	**< 0.001**	3.24 [2.53–4.55]	2.88 [2.47–3.67]	0.25	2.95 [2.47–3.85]	3.37 [2.87–3.52]	0.80
**Number of RCT published during the study period**	12 [5–21]	11 [4–20]	0.59	12 [5–21]	9 [5–20]	0.53	12.5 [6.00–23.25]	11 [3.25–20.00]	0.46	12 [5.0–21.0]	6 [5.0–9.0]	0.13
**Publisher (high vs. middle vs low)**			0.09			0.19			0.97			0.19
High	17 (41)	8 (20)		15 (28)	10 (34)		7 (29)	18 (31)		20 (26)	0 (0)	
Middle	14 (34)	22 (54)		21 (40)	15 (52)		11 (46)	25 (43)		32 (42)	4 (80)	
Low	10 (24)	11 (27)		17 (32)	4 (14)		6 (25)	15 (26)		20 (26)	1 (20)	
**Geographical area of the editorial committee board**			0.5			0.10			0.68			0.04
America	20 (49)	26 (63)		28 (53)	18 (62)		14 (58)	32 (55)		45 (58)	1 (20)	
Asia	3 (7)	3 (7)		6 (11)	0 (0)		2 (8)	4 (7)		4 (5)	2 (40)	
Europe	15 (37)	11 (27)		15 (28)	11 (38)		6 (25)	20 (34)		24 (31)	2 (40)	
Multinational	3 (7)	1 (2)		4 (8)	0 (0)		2 (8)	2 (3)		4 (5)	0 (0)	
**Journal topics**			0.44			0.44			1			0.33
Surgery in specialised topics	29 (71)	33 (80)		42 (79)	20 (69)		18 (75)	44 (76)		57 (74)	5 (100)	
Surgery in general topics	12 (29)	8 (20)		11 (21)	9 (31)		6 (25)	14 (24)		20 (26)	0 (0.0)	
**Publishing mode**			0.71			0.70			0.09			1.00
Reader	3 (7)	5 (12)		6 (11)	2 (6.9)		0 (0)	8 (14)		8 (10)	0 (0.0)	
Optional/Author	38 (93)	36 (88)		47 (89)	27 (93)		24 (100)	50 (86)		69 (90)	5 (100)	

Categorical data are reported with number and (%); continuous data are reported with median and [interval interquartile range (IQR)]. Significant *P* value (*P* < 0.0014) are highlighted in bold

*OIA* official instructions for authors, *ICMJE* International Committee of Medical Journal Editors

*Journals referenced as “Journals stating that they follow the ICMJE Recommendations” at http://www.icmje.org/journals-following-the-icmje-recommendations/

## Discussion

We noted low rates of implementation of data-sharing policies, i.e. 50% of the journals had no explicit policy included in their instructions for authors. When explicit, these policies were mostly optional contrary to the ICMJE recommendation that make a data-sharing statement mandatory for RCT. Of course, the study was performed during a time of change, and one could argue that the ICMJE position on data sharing was fairly recent (data extraction started 6 months after the ICMJE statement) and that a number of journals may not have sufficient time to implement it when our survey was conducted. However, this policy was announced in 2017 [4], and 35 (43%) journals are listed on the ICMJE website. Interestingly, the implementation of older policies was also suboptimal, even for making a specific reference to reporting guidelines such as CONSORT for RCT, which date from 1996 [6]. Except for COI disclosure, those recommendations were mostly non-binding. These disappointing results are not new. In 2014, Chapman et al. [10] warned about sub-optimal transparency policies in 10 leading surgical journals.

We considered a journal’s policies presented on its website as a surrogate marker of implementation of these policies. However, it is possible that editors of journals with a policy do not implement them in an optimal manner [11] or, conversely, that a journal with no specific policy documented on the website requires authors to comply with some of the features we explored. Of note, previous research has shown that journal requirements can have a significant impact on changing researcher practices [12], an obvious next step will be to explore the transparency features of the published RCT in these journals.

Of concern, we found no association of research transparency items with impact factors nor with the number of RCT published except for prospective trial registration among the surveyed surgical journals. This is of concern since impact factor is commonly used as a surrogate to assess the quality of a given journal and sometimes of an individual paper [13, 14].

Some limitations of our study have to be highlighted. Firstly, we want to stress that the goal of the study was to evaluate transparency research promotion by surgical journals through their official instruction for authors. Consequently, no conclusion about a relation between our results and the quality of published papers in terms of transparency can be drawn. Future research about journals or publishers’ policies’ impact on published papers will be important to assess the potential impact of such policies on reporting. Secondly, the official registration of our study protocol on OSF occurred after the initiation of the study, even though it was written before the review had begun and was and not modified since (author’s statement). The complete descriptive aspect of our main outcome limits the potential bias in this situation. Finally, we have chosen arbitrarily to combine mandatory and optional data-sharing policies based on the verbatim analysis we made during extraction. Data-sharing statement requirements were rarely mandatory but, in most cases, only optional. For exploratory analysis (journal features associated to research transparency promotion items), we combined these two categories in acknowledgment of the fact that journals suggesting data sharing is an important step in changing practice.

## Conclusion

Data-sharing policies appear to be sub-optimally adopted and promoted by surgical journals. We suggest that indicators of quality such as prospective audits of policies and published papers must be used to assess journals, instead of journal impact factors. We encourage surgical journals to improve their research transparency promotion. Standardisation of journal requirements according to ICMJE guidelines could be the first step forward for research transparency promotion in surgery.

## Data Availability

Study protocol is already available on Open Science Framework. Data extracted and statistical codes are available on Open Science Framework.

## Abbreviations

RCT: Randomised controlled trial
ICMJE: International Committee of Medical Journal Editors
COI: Conflict of interest
OIA: Official Instructions for Authors
IQR: Interquartile interval range
